# Phenotypic Plasticity of Yield and Yield-Related Traits Contributing to the Wheat Yield in a Doubled Haploid Population

**DOI:** 10.3390/plants13010017

**Published:** 2023-12-20

**Authors:** Md Atik Us Saieed, Yun Zhao, Kefei Chen, Shanjida Rahman, Jingjuan Zhang, Shahidul Islam, Wujun Ma

**Affiliations:** 1 Food Futures Institute, School of Health, Education & Environment, Murdoch University, Perth, WA 6150, Australia; 2 Department of Seed Science & Technology, Bangladesh Agricultural University, Mymensingh 2202, Bangladesh; 3 Curtin Biometry and Agriculture Data Analytics, Molecular and Life Sciences, Curtin University, Bentley, WA 6102, Australia; 4 Department of Genetics & Plant Breeding, Bangladesh Agricultural University, Mymensingh 2202, Bangladesh; 5 Department of Plant Sciences, North Dakota State University, Fargo, ND 58108, USA; 6 College of Agronomy, Qingdao Agriculture University, Qingdao 266109, China

**Keywords:** phenotypic plasticity, grain yield, segregation analysis, wheat

## Abstract

Phenotypic plasticity is the ability of an individual genotype to express phenotype variably in different environments. This study investigated the plasticity of yield-related traits of bread wheat by utilising 225 doubled haploid (DH) lines developed from cv. Westonia and cv. Kauz, through two field trials in Western Australia. Plasticity was quantified via two previously published methods: responsiveness to varying ecological conditions and slopes of reaction norms. The spikelets/spike was the most plastic trait, with an overall plasticity of 1.62. The least plastic trait was grain protein content, with an overall plasticity of 0.79. The trait hierarchy based on phenotypic plasticity was spikelets/spike > thousand kernel weight > seed number > seed length > grain yield > grain protein content. An increase in yield plasticity of 0.1 was associated with an increase in maximum yield of 4.45 kg ha^−1^. The plasticity of seed number and grain protein content were significantly associated with yield plasticity. The maximal yield was positively associated with spikelets/spike and grain yield, whereas it negatively associated with grain protein content. In contrast, the minimal yield was found to be negatively related to the plasticity of spikelets/spike and the plasticity of grain yield, whereas it was not related to grain protein content plasticity. Seed number and seed length exhibited plastic responses at the higher fertilisation state while remaining relatively stable at the lower fertilisation state for the wheat DH population. The finding of the current study will play a key role in wheat improvement under the changing climate. Seed length and seed number should be the breeding target for achieving stable yield in adverse environmental conditions.

## 1. Introduction

Phenotypic plasticity refers to the expressional flexibility of a particular trait in response to external factors, such as changes in environmental conditions [1]. It is the genetic regulation of ecological influences on the trait. Phenotypic plasticity varies among traits, genotypes, and environments with various fitness effects [1,2]. Many organisms express different phenotypes under different environmental conditions. Phenotypic plasticity allows organisms to express a trait that better fits their particular environment [3]. The significance of phenotypic plasticity depends on the specific situation [1,4,5]. Cultivars with stable performances under stressed environmental conditions are appreciated for their expected and reliable yields. On the contrary, stability is not well-accepted under controlled ecological conditions as plants cannot take advantage of favourable environmental conditions. Plants that are unable to migrate must cope with environmental heterogeneity. Plastic responses that plants may produce under differential ecological conditions may interfere with the plant breeding target, making the process critical. Due to plastic responses, plant breeders are adopting several strategies in breeding [6]. There is a chance for breeders to exploit plasticity to produce high yields in favourable environments and lower products in poor environments [4,6].

Plasticity could be influenced by genetic factors, such as single-nucleotide polymorphisms (SNP) or a variation caused by duplication and polyploidy [7]. In these cases, gene redundancy and copy number variation (CNV) might affect the level of produced transcripts for more continuous phenotypic responses under flexible environmental conditions [8]. The epigenetic control of gene expressions, such as DNA methylation and histone modification, might also be responsible for the variation in plastic responses [9].

Plasticity and stability are inversely related. As such, a high level of plasticity indicates low stability and vice versa. T. Roemer first coined the term “stability” in 1917. There are two types of stability: (1) static stability, which denotes a slight variance in phenotypic expression level exhibited by a genotype under different environmental conditions; (2) and dynamic stability or equivalence, which refers to phenotypic responses that vary predictably among environments, but all genotypes are affected equally [10].

It is well-understood that phenotypic variation occurs due to the interaction effect of the genotype and environment [11]. Wheat grain yield and its components are largely genetically controlled, and environmental conditions significantly influence them [12,13]. The phenotypic response is not the same for all genotypes to changing environments. Strong G × E interaction often positively and negatively affects the different traits and expressions. Plant breeding is inherently complex under various environmental conditions. Recommending the best cultivars for adaptation or higher yield in an untested environment becomes challenging due to the interaction effect [14,15]. Other yield components might partially compensate for the failure to form a yield component, which is a common phenomenon in plants. Therefore, yield stability is achieved due to the contribution of the compensation ability [16]. Crop improvement depends on understanding, quantifying, and exploiting the genotype–environment interaction effect [1,17,18,19]. The phenotypic plasticity of plants regulates their behaviour under changing environmental conditions by adapting their morphological, physiological, and phenological properties [20,21]. The survival of plants depends on the phenotypic changes, which are essential and related to plant fitness under different climatic conditions [22]. Thus, quantifying the plasticity of the trait of interest is crucial to planning a breeding program.

Different traits and their plasticity seem inter-related, indicating that the plasticity of a specific trait can influence that of others. This inter-relation can be seen, for example, between the plasticity of grain yield and that of spike number [23], and between yield plasticity and the plasticity of time to anthesis [24]. The plasticity of yield components in cereal was ranked by Sadras et al. [25], where the highest plasticity was obtained in the tiller number, whereas grain weight plasticity was the lowest [25]. Reduced grain number plasticity was also observed in reducing the inflorescence number, which is genetically controlled. The introduction of *tin* alleles in wheat, which reduces tillering, was associated with the increased plasticity of grain weight [26]. In wheat, a high yield is favoured by high grain numbers, which can be achieved by producing either many small spikes or fewer, more enormous spikes [27]. The plasticity of several traits of maize hybrids in response to plant population density was ranked by Bonaparte and Brawn [28]. Among them, grain yield was the most plastic, and the least plastic were ear number and ear height. Bradshaw [1] proposed that trait hierarchy results from a conditional relationship indicating that the stability of a given trait is “at least in part the outcome of the plasticity of the other characters”. Therefore, seed number plasticity is a condition for the stability of seed size [25]. The plasticity of different traits can be arranged into a hierarchical order focusing on the trade-offs which maximize genetic fitness [1]. It will be easier to understand the relationships among traits through the hierarchies [26,29]. Trait hierarchies may also provide insight into the environmental responses of the related traits.

Plasticity can be quantified by the slope of norms of reaction where statistical approaches are necessary to relate phenotypic traits to the environmental variable [17]. Understanding plants’ response to the changing environment will improve the present knowledge of plant adaptation and links between phenotype and genotype [24,30]. Finley and Wilkinson [31] used the linear regression coefficient between the genotype mean in each environment and the overall mean for each atmosphere to determine stability parameters while other researchers used it to estimate the plasticity of crop response [24,32].

The recent improvement in plant molecular biology approaches in identifying the quantitative trait locus (QTL) for the trait of interest has drawn enormous attention, followed by marker-assisted selection breeding. Accordingly, many QTLs have been identified and integrated into breeding programs. However, a significant improvement of traits under field conditions has not been achieved consistently because of the lack of understanding of the traits’ phenotypic plasticity and the plasticity interaction dynamics among multiple attributes. Identifying the genetic loci of a particular trait is generally performed based on the phenotypic variations of the specific traits across the structured population. The contribution of the genetic component to these phenotypic variations is influenced by trait plasticity, which is crucial to predicting breeding efficiency. In addition, a significant plasticity interaction between two traits might indicate whether or not both characters should be considered in the breeding. In particular, characterising the trait plasticity of the target trait and the associated characteristics and interaction will give confidence in the breeding program. The study was designed from a wheat DH population developed from crosses between Westonia and Kauz [33]. This study evaluated the variation in yield-contributing traits, estimated traits’ plasticity and interrelation and determined the basis of trait plasticity being favoured by grain yield or grain protein content.

## 2. Materials and Methods

### 2.1. Plant Materials

The experiment consisted of 225 doubled haploid (DH) lines developed from Westonia × Kauz. The wheat cultivar Westonia is an Australian cultivar, with a high yield capacity in medium- and low-rainfall conditions. The cultivar Kauz is industrialised by the International Maize and Wheat Improvement Center (CIMMYT), containing 1B.1R translocation with a higher grain weight and more water-soluble carbohydrates than Westonia in well-watered conditions [34].

### 2.2. Field Trials

Two field trials were carried out in the wheat season of 2016. One was performed at the University of Western Australia’s research field station located at Shenton Park, Western Australia (32° S 115.9° E), and the other was at Wongan Hills, Western Australia (30.9° S, 116.7° E), following a randomised complete block design (RCBD). The meteorological data of the season are presented in Table S1. At Shenton Park, among the 225 DH lines, 152 DH lines were selected based on the availability of seeds and planted in a 1 × 0.67 m^2^ plot with three nitrogen fertilisation rates (0, 50, and 100 Kg/ha), using urea–ammonium nitrate (UAN) as the N source. At Wongan Hills, among the 225 DH lines, 143 DH lines were selected based on the availability of seeds and planted in a 2 × 0.6 m^2^ plot with two doses of nitrogen fertilisation (50 and 100 Kg/ha). Two replications were used for 76 DH lines and 71 DH lines at Shenton Park and Wongon Hills, respectively, and the rest of the DH lines at both locations had no replication. Nitrogen fertilizer was applied at the stem elongation stage. Insecticides and herbicides were regularly sprayed to control insects and weeds.

### 2.3. Phenotyping

Data on phenotypic and yield-related traits were collected, including thousand kernel weight, seed length, spikelets/spike, spike number, grain yield, and grain protein content. Phenotypic data were obtained using an image analysis system (SeedCount Machine, Next Instruments Pty Ltd., Condell Park, NSW, Australia). For the investigation of yield component (thousand kernel weight, spikelet number per spike, seed length, and seed number) traits, five uniform plants were selected from each plot. The remaining were harvested for yield measurement. Grain protein content was measured with the FOSS NIRS XDS instrument (FOSS NIR Systems, Silver Spring, Haymarket, NSW, Australia).

### 2.4. Estimation of Plasticity

A two-step ordinary least-squares (OLS) procedure was used to estimate the phenotypic plasticity [35]. The first step was used to estimate the environmental effect as a main effect with the model as follows:

(1) $$y_{ij}=\mu +g_{i}+h_{i}+\varepsilon_{ij}$$

where $g_{i}$ is the main effect of ith variety, $h_{j}$ is the main effect of the jth environment, and $\varepsilon_{ij}$ is an error term with a normal distribution $\left[\varepsilon_{ij}\sim N\left(0,\;\delta^{2}\right)\right]$.

The second step is used to estimate the intercepts and slopes of each variety/line with the model as follows:

(2) $$y_{ij}=\mu +g_{i}+(b_{i}+1)\cdot {\hat{h}}_{j}+\varepsilon_{ij}$$

where ${\hat{h}}_{j}$ is the estimate of environmental effect from step 1, and $b_{i}+1$ is the change in the expected variety performance per unit change in the environment effect (${\hat{h}}_{j}$).

The coefficient of phenotypic plasticity was derived as the dimensionless slope of the linear regression between the trait of an individual line and its mean in that environment [31]. Thus, a slope equal to 1 indicates average performance in all environments, a slope greater than 1 indicates above-average performance, and a slope less than 1 indicates below-average performance. Reaction norms describe phenotypic plasticity in several ways, such as cross-over interaction (where reaction norms cross with each other) or scale effect (SC) interaction (no intersection between reactions norms). Cross-over interaction implies a change of genotypes across the environmental range [21,36]. The overall plasticity was estimated by taking the average of all the DH lines for each trait.

### 2.5. Joint Segregation Analysis

The inheritance analysis used the joint segregation analysis method [37,38]. Joint segregation analysis utilizes an expectation and iterated maximisation (EIM) algorithm to obtain the maximum likelihood estimators (MLEs) of parameters in the joint segregation analysis [38]. In total, 18 types of inheritance models were tested for the DH population. These inheritance models can be classified into seven groups: the one major gene model (A), two major gene model (B), pure polygene model (C), one major gene plus polygene model (D), two major gene plus polygene model (model E), three major gene model (F), three major gene plus polygene model (G), four major gene model (H), and four major gene plus polygene model (model I). Model selection was conducted by using Akaike’s information criterion (AIC) [36], the likelihood ratio test, and a series of goodness-of-fit tests including the uniformity test ($U_{1}^{2}$, $U_{2}^{2}$, and $U_{3}^{2}$), Smirnov test (_n_W^2^), and Kolmogorov test (Dn) [37]. The estimates of genetic parameters, including the additive, dominance, and epistasis effects of the major gene(s), the total additive, dominance, and epistasis effects, genetic variances for major genes and polygenes, and the heritability of major genes and polygenes, were obtained from the best-fitted genetic models for each phenotypic trait and environment.

## 3. Results

### 3.1. Phenotypic Plasticity of Yield-Related Traits

The phenotypic plasticity ranged from 5.168 to −2.888 based on different traits (Table 1). The ranges for trait plasticity were greater for thousand kernel weight, spikelets/spike, and seed number, whereas the ranges were narrow for other traits including grain yield, grain protein content, and seed length (Table 1). The average trait plasticity was the highest for the spikelet number/spike, which was 1.62, and the lowest was for grain protein content which was 0.79 (Table 1). The plasticity of different traits varied from genotype to genotype. Each genotype also exhibited a variation in plasticity depending on the traits (Table S2). Average trait plasticity considering all genotypes was close to 1 for some traits, such as seed number and length (Table 1). A trait plasticity of near one or greater indicates plants’ ability to benefit from favourable environmental conditions. The trait hierarchy based on phenotypic plasticity was spikelets/spike > thousand kernel weight > seed number > seed length > grain yield > grain protein content.

### 3.2. Influence of Yield Plasticity on Yield

**Figure 1:**
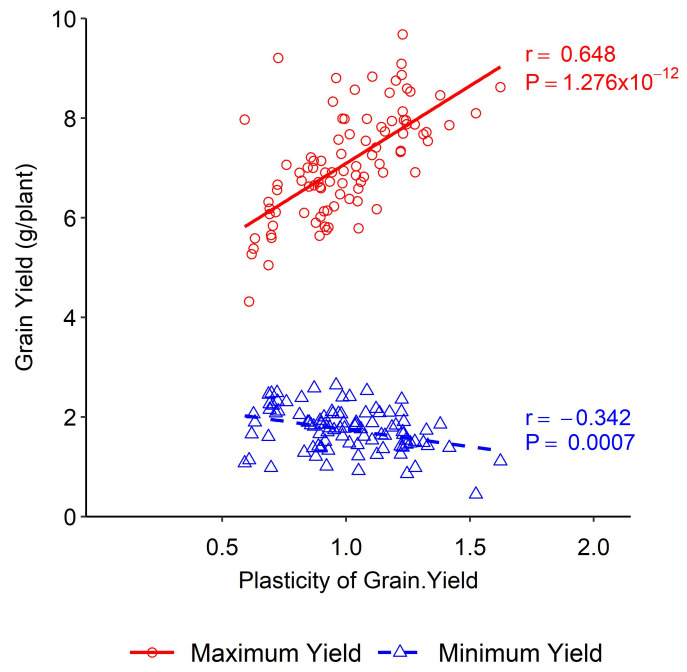
Association between phenotypic plasticity of grain yield and minimum and maximum yield in wheat DH lines.

**Figure 2:**
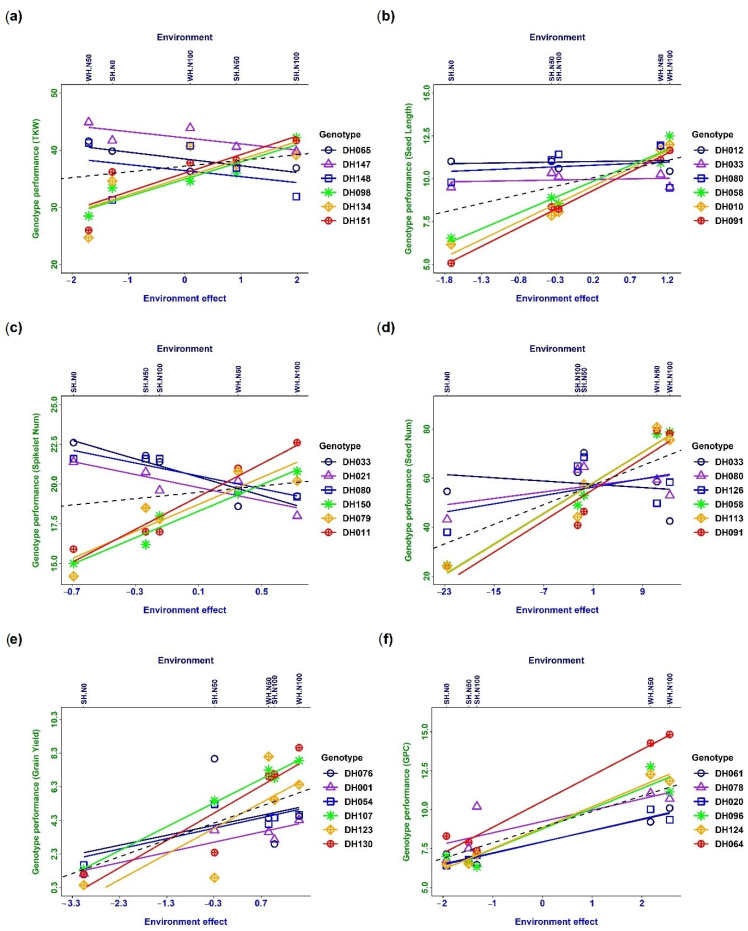
Reaction norms of phenotypic plasticity and genotype–environment interaction (G × E) for different traits: (**a**) thousand kernel weight, (**b**) seed length, (**c**) spikelet number per spike, (**d**) seed number, (**e**) grain yield, and (**f**) grain protein content. The dashed line indicates slope = 1. Only three genotypes with the highest and lowest slopes are plotted.

A positive association was obtained between yield plasticity and maximum yield under different nitrogen levels, whereas a negative association was obtained between yield plasticity and the minimum yield. Higher yield plasticity was obtained at higher nitrogen doses under both experimental conditions (Figure 1). High yield plasticity was associated with the capacity to benefit from favourable conditions rather than with responsiveness to poor environments (Figure 2e).

### 3.3. Influence of Other Traits’ Plasticity on Yield

**Figure 3:**
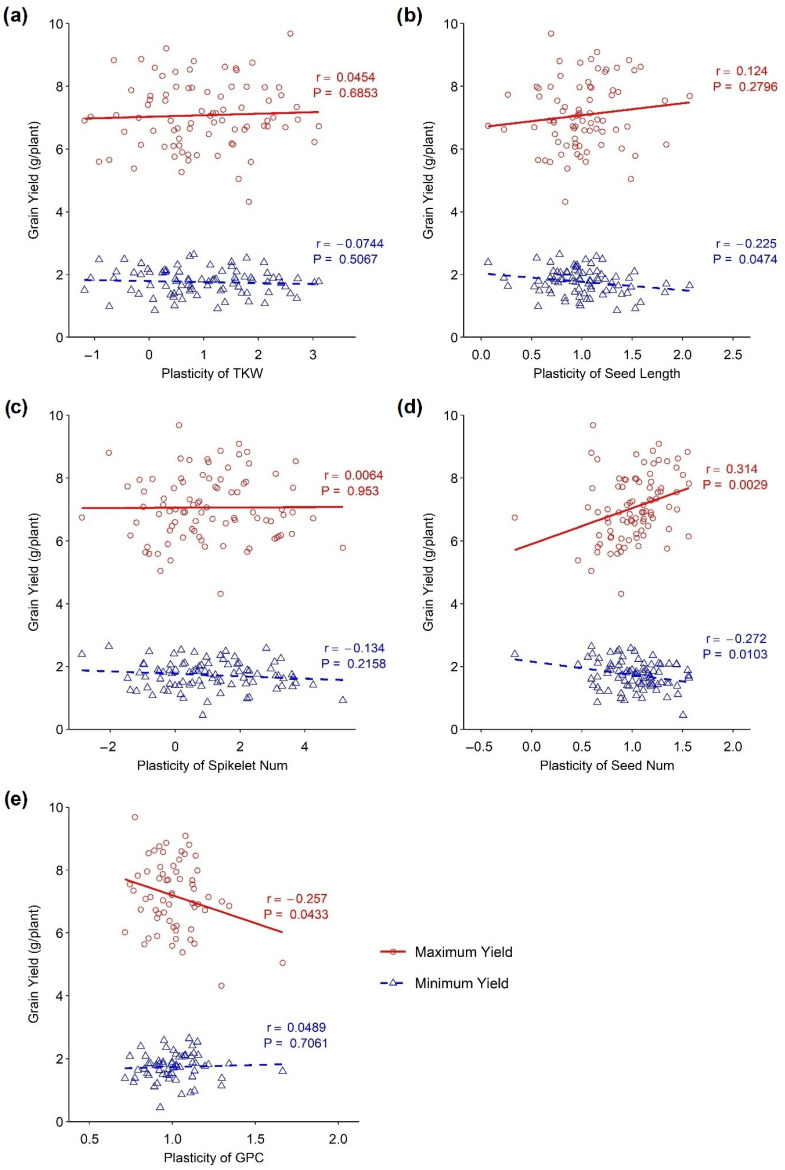
Association between phenotypic plasticity of yield-related traits and minimum and maximum yield in wheat DH lines. (**a**) Thousand kernel weight, (**b**) seed length, (**c**) spikelets/spike, (**d**) seed number, and (**e**) grain protein content.

The associations of the plasticity of different traits with a maximum and minimum yield under different environmental conditions are presented in Figure 3. A positive association between the maximum yield and the plasticity of yield components, such as seed length and seed number, has been observed. A significant positive association was observed between the plasticity of seed number and maximum yield (*p* < 0.001, r = 0.314), whereas there was no significant association between seed length and maximum yield. On the other hand, a negative association was observed between the plasticity of grain protein content and maximum yield, which was significant (*p* < 0.001, r = −0.257). Similarly, there was no association between the plasticity of the thousand kernel weight and spikelet number/spike with the minimum and maximum yield as demonstrated by the slope, which is close to zero (Figure 3). A negative but significant association was observed between the minimum yield and the plasticity of seed length (*p* < 0.05, r = −0.225) as well as between the minimum yield and the plasticity of seed number (*p* < 0.05, r = −0.272). Again, no significant association was identified between minimum yield and the plasticity of grain protein content.

### 3.4. Trait Plasticity and Genotype–Environment Interaction

The phenotypic response of different traits to the changing environments is expressed as reaction norms, and G × E interaction occurs when the slope of the reaction norms differs for different DH lines (Figure 2). Cross-over interaction meaning reaction norms cross each other was observed in the case of thousand kernel weight (Figure 2a), spikelet number per spike (Figure 2c) and seed number (Figure 2d). Only a few extreme genotypes are plotted for the better observation of cross-over interaction. The results indicated a significant genetic effect on the phenotype along with environmental interaction. A scale effect (SC) interaction indicated no intersection between reaction norms, which were observed for seed length (Figure 2b), grain yield (Figure 2e), and grain protein content (Figure 2f). The observation implies that the changes in the phenotype were mainly due to the environment, where the genetic difference among wheat DH lines did not have much influence on the genotypes.

### 3.5. Plasticity in Determining Traits’ Interrelationships

**Figure 4:**
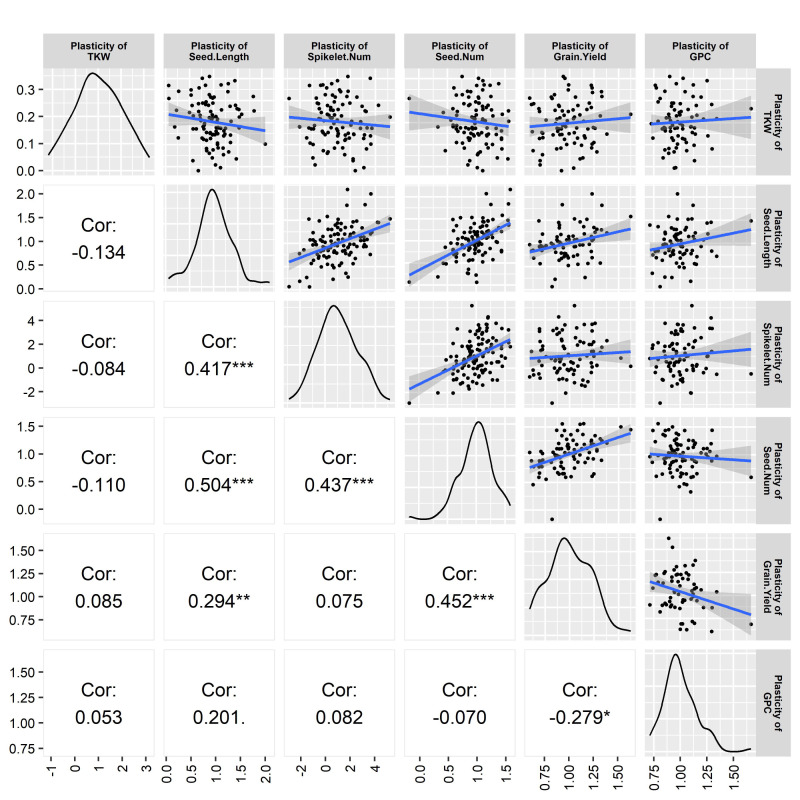
Correlation among different trait plasticities. The bell curve and scatter plots indicates the quality and distribution of the data. Values are Pearson correlation coefficients and significance level indicated by * p<0.05, ** p<0.01, *** p<0.001.

The plasticity of different traits seemed to be interrelated, as observed in the correlation analysis. Seed length plasticity exhibited a significant and positive relationship with the plasticity of spikelet number per spike (medium correlation, *p* < 0.001), seed number plasticity (high correlation, *p* < 0.001), and the plasticity of grain yield (low correlation, *p* < 0.05). Seed number plasticity showed a significant (*p* < 0.001) and positive relationship with the plasticity of spikelet number per spike (medium correlation) and grain yield plasticity (medium correlation). Grain yield plasticity had a significant relationship (*p* < 0.05) with the plasticity of grain protein content, which was low and negative (Figure 4).

### 3.6. Joint Segregation Analysis of the Genes Governing Grain Yield and Yield-Related Traits

Joint segregation analysis of the genes regulating different traits revealed that all traits were polygenic, and the number of genes varied depending on the traits. However, analysis of the phenotypic data suggested that the traits were primarily regulated by two to four genes (Table 2). The cumulative effects of the genes of the corresponding traits were variable. For example, the genetic model 4MG-EEA was found to be most suitable for grain yield, meaning four significant genes control the feature and partially equal additive where d_a_ = d_b_ = d_c_ = d_1_, and d_d_ = d_2_, indicating that the trait was the outcome of the expression of two groups of genes, where, gene 1, 2 and 3 formed group 1 and had an equal and additive effect whereas, gene 4 belonged to group 2 for which effect level was different from that of the other three. Significant genetic variation indicated the proportion of the trait explained by the primary gene, ranging from 0.02 to 23.07 of the total phenotypic variation, which was dependent on the trait and influenced by the environment. The heritability of the traits for the significant genes was medium to high except for that of grain protein content and varied from 6.5 to 95.37% (Table 2). The detailed result is available in Supplementary File S1.

Notations of model codes:

1. 1MG ~ 4MG: One to four major genes.
2. AE: *d_a_* = *d_b_*, additive effect with interactions between the two major genes.
3. ER: epistatic recessiveness of aa on Bb locus.
4. EA: *d_a_* = *d_b_*, equally additive, not considering the interaction between the two major genes.
5. AI: Additive epistasis.
6. CEA: *d_a_ = d_b_ = d_c_ = d*, completely equally additive.
7. PEA: *d_a_ = d_b_ = d*_1_*, d_c_ = d*_2_, partially equally additive.
8. EEA: *d_a_ = d_b_ = d_c_ = d*_1_, *d_d_ = d*_2_, partially equally additive.
9. EEEA: *d_a_ = d_b_ = d_1_*, *d_c_ = d_d_ = d*_2_, partially equally additive.

Only five-grain yield QTLs were identified in a previous study using the Westonia–Kauz DH population, considering different environmental conditions [39]. The associated chromosomes were 1B, 5A, 7A and 7D. The segregation analysis mostly detected the four causal genes depending on the environmental conditions, though the interaction effect among these genes was diverse. However, the major gene variance was very low, indicating other unexplored regulating factors. The phenotypic plasticity played a major role here. Yield plasticity was found to be moderate among the yield-contributing traits. Additionally, genes regulating this important trait exhibited an additive interaction effect. The influence of the QTL on trait plasticity needs to be explored further.

## 4. Discussions

### 4.1. Quantification and Comparison of Plasticity of Yield and Yield Components

The trait plasticities were plotted in two different regimes considering the maximum and the minimum yield level, which were found to be different based on the maximum- and the minimum-yield conditions, indicating that plastic performances were environmentally dependent. Statistical models of phenotype vs. environment are widely used to estimate phenotypic plasticity. The models can be linear or non-linear [40,41], stating that the phenotype versus environment models are robust if the vital environmental drivers are known and measured. This study used a simple phenotype vs. environment model to quantify phenotypic plasticity. The coefficient of phenotypic plasticity was derived as the dimensionless slope of the linear regression between the trait of an individual line and its mean in that environment [31]. The degree of plasticity depends on the differences in phenotypic changes over changing environments. The phenotypic changes near zero represent a stable phenotype, whereas, apart from zero, they indicate a plastic phenotype [1,4]. Accordingly, two types of association between the maximum and the minimum yield and the plasticity of yield components were observed under differential environmental conditions (Figure 1 and Figure 2).

High yield plasticity refers to the ability of a plant to produce a high yield under varying environmental conditions. This trait is more closely associated with the plant’s ability to take advantage of favourable conditions rather than its ability to respond to poor environments. This means plants with high yield plasticity are more likely to thrive when conditions are good rather than simply surviving when conditions are poor. This has important implications for crop breeding, as it suggests that selecting for high yield plasticity may be more effective at improving crop yields than is selecting for stress tolerance alone. Environments with high nitrogen fertilisation favoured wheat yield, meaning plants could utilise the environmental stimulus, whereas, in the case of lower nitrogen fertilisation, the wheat yield was decreased. Still, the rate of decrease was not proportional to the increase under favourable conditions. This indicated that the yield plasticity was less in unfavourable conditions. In the Westonia × Kauz DH lines, an increase in plasticity of 0.1 units was associated with an increase in maximum yield of 0.3 g/plant or 4.45 kg ha^−1^. In the case of minimum yield, an increase in plasticity of 0.1 units was associated with an increase of 0.06 g/plant or 0.9 kg ha^−1^, respectively (Figure 1). High yield plasticity was agronomically valuable under favourable environmental conditions as it was associated with the capacity to benefit from good conditions. Low yield plasticity was beneficial for unfavourable climatic conditions as it produced stable yield in poor environments. In our study, yield plasticity for the maximum yield ranged from 0.5 to 1.6 and for the minimum yield it ranged from 0.9 to 1.6. Such differences in yield plasticity have agronomic significance. When yield plasticity is higher for a breeding line, farmers might expect a better yield in favourable environmental conditions [42]. As mentioned earlier, the numbers of the identified QTLs for grain yield were few. Additionally, a single stable QTL was detected in various environments. The genetic dissection of this important QTL is needed for updating the current understanding of yield plasticity and its incorporation into genetic improvements in wheat.

The seed number is the primary component of grain yield, determining wheat’s final product. In this study, the plasticity of seed number was positively associated with the maximum yield indicating that a higher yield was achieved as the trait became more plastic, whereas seed number plasticity was negatively associated with the minimum yield, indicating that reduced yield was achieved due to the stability of the trait. The average performance indicated plants’ ability to perform similarly under different environmental conditions. While high yield plasticity may be associated with the capacity to benefit from favourable conditions, seed number plasticity may still perform above average in response to poor environmental conditions. Seed number plasticity can be a more effective trait in responding to poor environments. In our study, only a few lines showed above-average seed number plasticity in response to limited plant resources or adverse environmental conditions. This suggests that selecting seed number plasticity may be more effective at improving crop yields under poor environmental conditions. In other words, this trait could utilise available resources for better performances under favourable conditions. In the shortage of resources, seed numbers became stable, which is crucial for a steady yield. Bradshaw [1] argued that grain number plasticity is a condition for seed size stability, as supported later by Sadras and Trentacoste [30]. Cheplick [43] also observed that seed number was significantly plastic in perennial grass.

On the other hand, the plasticity of grain protein content exhibited a significant negative association with maximum yield, whereas no association was obtained in the minimum yield. Nitrogen is the primary component of both grain protein and yield. The partitioning of this essential macronutrient was the determining factor for these competing yield components. It is a common phenomenon that sacrificing one of the components results in the better performance of the other. Increasing the plasticity of the grain protein content resulted in a reduced grain yield. A similar observation was found by Peltonen-Sainio [42], who found that high yield plasticity is associated with low grain protein content.

Thousand kernel weights exhibited a range of plasticity. Still, it was not associated with the minimum and maximum yield (Figure 3a). It might happen that the plasticity of thousand kernel weight did not directly affect the grain yield. Probably, it interacted with the other yield components to influence the grain yield. Some DH lines with high plasticity for this trait exhibited poor performances under adverse environmental conditions. In contrast, they also showed better performances under favourable conditions (Figure 2a). Yarosh et al. [44] stated that kernel weight in rapeseed had very high plasticity, compensating for kernel number reduction. The component characters shifted in a compensating manner in the changing environments to provide a stable yield [45,46].

All the traits exhibited some level of plastic responses, though the range of plasticity varied depending on the traits. This phenomenon might be due to the differential expression behaviour of the traits regulating genes and their interaction effects. Environments also played a crucial role in influencing gene expression. Higher trait plasticity indicated stronger genetic regulation of the traits in the interacting environments. In comparison, low trait plasticity might happen due to genes’ insensitivity to the environmental stimulus or the presence of fewer interactions of the trait-regulating genes.

### 4.2. Genotype–Environment Interaction on Yield and Related Traits

Phenotypic plasticity refers to the ability of an organism to exhibit different phenotypes in response to different environmental conditions, without any change in its genetic makeup. Moreover, phenotypic plasticity is a characteristic and developmental stage-specific trait. This means that the level of plasticity can vary depending on the developmental stage of the organism and the specific characteristic being measured [1]. Reaction Norms express the phenotypic response to the environment (Figure 2). The slope of the reaction norms differs for different genotypes. Percentile–plasticity plots (Figure 1 and Figure 2) are often used for comparing plastic responses among traits, though reaction norms provide a better understanding of trait plasticity in changing environments. Cross-over interaction was observed in combination with several genotypes, indicating that genotype–environment interaction happened. A strong interaction was observed for thousand kernel weight, spikelet number per spike, seed number, and grain yield (Figure 2). These traits are under strong genetic regulation. The trait-regulating genes also interacted among themselves and were influenced by the environmental stimulus. In contrast, it was observed that seed length and grain protein content were not significantly affected by the environment (Figure 2). This may be due to environmental canalisation, which is the ability of a trait to remain relatively stable in the face of environmental variation. Alternatively, it may be possible to trade off some traits, such as seed length and number, to achieve better performance in other characteristics. Furthermore, yield components were found to be negatively related to each other, indicating competition for resources in response to environmental variation. However, this competition between grain number per square metre and thousand kernel weight was absent. This suggests that different yield components may be influenced by different environmental factors and that selecting for specific traits may be more effective than selecting for overall yield. By understanding these relationships and trade-offs, we can develop more effective breeding strategies to improve crop yields in changing environments [25]. It was observed that seed length and grain protein content were not significantly affected by the environment (Figure 2). This may be due to environmental canalisation, which is the ability of a trait to remain relatively stable in the face of environmental variation. Alternatively, it may be possible to trade off some traits, such as seed length and number, to achieve better performance in other characteristics.

Furthermore, yield components were found to be negatively related to each other, indicating competition for resources in response to environmental variation. However, this competition between grain number per square metre and thousand kernel weight was absent. This suggests that different yield components may be influenced by different environmental factors and that selecting for specific traits may be more effective than selecting for overall yield. By understanding these relationships and trade-offs, we can develop more effective breeding strategies to improve crop yields in changing environments. Grafius [46] presented an analysis of such interrelationships of yield components in cereals where he suggested that the breeder should not ignore principles of balance among the components.

### 4.3. Correlation between Yield Plasticity and Another Trait’s Plasticity

The correlation analysis of trait plasticity exhibited that grain yield plasticity is highly significant and positively correlated with the plasticities of seed length and seed number, indicating that the plasticity of these yield components heavily contributes to grain yield plasticity. Again, the plasticity of seed number significantly correlated with the seed length plasticity and the plasticity of the number of spikelets/spike (Figure 4). The plasticity of seed length significantly correlated with the plasticity of the number of spikelets/spike. The correlation analysis indicated the interaction of contributing genes for phenotypic expression. All the traits were interrelated, as well as were the genes regulating the traits. The plasticity of some traits directly influenced the grain yield plasticity. Furthermore, the plasticity of some other traits indirectly influenced the grain yield plasticity, as they directly influenced the plasticity of yield-related traits.

The interrelation among the yield and yield components indicates their flexibility and the compensation of one yield component by others, where seed number, seed length, and spikelets/spike are the major contributing factors for grain yield. A significant positive relation between seed number plasticity and yield might have a dominating role in yield determination. In the case of annual and perennial crops, negative and positive correlations between yield and agronomic traits were also common [1,24]. The plasticity of grain yield and protein content exhibited a significant negative correlation. Peltonen-Sainio [42] observed that high yield plasticity was associated with low grain protein content and cereal cultivars with high yield potential, prone to high yield variability but low grain protein content.

### 4.4. Genes Controlling Different Traits and Their Effect

In nature, most of the characteristics are polygenic, meaning that multiple genes are responsible for each trait. The combined gene effects, such as the expression levels of all complex and ambiguous genes, provide the final indication of a characteristic. Redline [47] defined segregation analysis as a statistical approach where assumptions of genetic models on the mode of inheritance are compared along with the impact of environmental factors to identify the most suitable model for explaining the underlying distribution of traits. For this purpose, the maximum likelihood function was established from a single segregating generation. The procedure included and estimated the proportion, mean, and variance of each component distribution from the experimental data. Seven types of genetic models have been formed: one major gene model (model A), two major gene model (model B), pure polygene model (model C), one major gene plus polygene model (model D), two major gene plus polygene model (model E), three major gene model (model F), three major gene plus polygene model (model G), four major gene model (model H), and four major gene plus polygene model (model I). There were several models regarding the additive, dominance, and epistasis effect of significant genes and polygene in various segregating generations in each type. The iterated ECM (IECM) algorithm was used to estimate the number of component distributions in a mixture distribution. Akaike’s information criterion (AIC) [48] and a uniformity test (U^2^), Smirnov test (nW^2^), and Kolmogorov test (Dn^2^) [49] were used to choose an optimal genetic model and pick up its corresponding estimates of component distributions. The estimates of genetic parameters, including the additive, dominance, and epistasis effects of the major gene(s), the total additive, dominance, and epistasis effects, and heritability of the major gene, and the heritability of the polygene, were calculated from the estimates of the component distributions of the optimal genetic model using the method of least squares according to the relationship between the genetic parameters and the component distribution parameters of a given model [37,38,50,51].

The results obtained from such analysis show the number of genes responsible for expressing a specific feature and their interaction in different environments. However, identifying responsible genes cannot be achieved from such an analysis. Several associations among the genes are also present. The associations include additive, overdominance, and pleiotropy or allelic sensitivity, epistasis, epigenesis, and so forth [36]. Segregation analysis was performed to observe the number of genes responsible for yield and yield-related characteristics and their association. The genetic model containing two to four significant genes was observed to be most appropriate for all the traits. However, the association of these genes was found to be different under different environmental conditions. Mainly, these genes exhibited additive effects for the expression of each trait, though the type and amount of addictiveness were found to be diverse. This indicated the genotype–environment interaction for the phenotypic expression. The association among the genes varied along with the environmental variation, which was ultimately reflected in the plasticity of different traits. The major gene variance was very low for all the traits, revealing the presence of unidentified factors or genes. As the transcription and translation of genes are also involved in phenotypic expression, these should be included in the analysis to understand the phenotypic plasticity of the traits better. Zhao [33] performed QTL mapping for wheat DH lines for kernel weight and nitrogen use efficiency-related traits and obtained the association of multiple genes, i.e., polygenic control over the essential characteristics.

## 5. Conclusions

A better understanding of plasticity is crucial in plant improvement under rapid climate change. High yield plasticity is expected for intensively managed growing conditions, as it is associated with the capacity to benefit from favourable conditions. Low yield plasticity might be necessary for adverse and lowly managed growing conditions. Though the spikelets/spike was the most plastic trait, the trait plasticity did not directly influence the grain yield plasticity. Furthermore, high yield plasticity tends to be positively associated with the plasticity of seed length and number but tends to be negatively associated with the plasticity of grain protein content. Thus, breeders should focus on seed length and number to achieve a stable yield. Again, the environment greatly influences the number of genes and their interaction in phenotypic expression. It is necessary to find out the causal gene(s) in trait formation and their incorporation into wheat breeding for the sustainable improvement of wheat grain yield.

## Figures and Tables

**Table 1:** Range of phenotypic plasticity for different traits and average plasticity.

Phenotypic Trait	Range of Trait Plasticity		
	Maximum	Minimum	Overall
Thousand kernel weight	3.247	−1.186	1.23
Seed length	2.165	0.063	0.92
Spikelets/spike	5.168	−2.888	1.62
Seed number	1.593	−0.166	0.93
Grain yield	1.623	0.59	0.81
Grain protein content	1.664	0.713	0.79

**Table 2:** Segregation analysis of grain yield and yield-related traits in different environments.

Trait	Environment	Selected Genetic Model	Major Gene Variance	Heritability (Major Gene) (%)
Thousand kernel weight	SH.N0	2MG-AE	4.438	32.94
	SH.N50	2MG-ER	1.087	7.69
	SH.N100	4MG-EEEA	4.063	33.73
	WH.N50	4MG-EEEA	23.07	95.37
	WH.N100	4MG-EEEA	9.368	55.87
	All env.	4MG-CEA	2.653	47.43
Seed length	SH.N0	3MG-CEA	0.539	52.55
	SH.N50	4MG-EEEA	0.174	29.96
	SH.N100	4MG-EEA	0.796	79.88
	WH.N50	4MG-EEA	0.564	76.87
	WH.N100	4MG-EEA	0.256	36.65
	All env.	4MG-EEEA	0.703	95.33
Spikelets/spike	SH.N0	4MG-EEEA	0.107	68.47
	SH.N50	4MG-EEEA	1.829	72.00
	SH.N100	4MG-EEA	0.488	22.76
	WH.N50	4MG-EEEA	0.955	56.47
	WH.N100	4MG-EEA	1.533	93.40
	All env.	4MG-EEA	0.417	77.53
Seed number	SH.N0	3MG-AI	3.944	9.606
	SH.N50	3MG-A	3.227	6.655
	SH.N100	4MG-EEEA	4.246	26.21
	WH.N50	3MG-AI	5.506	8.162
	WH.N100	3MG-A	4.547	6.492
	All. Env	4MG-EEEA	5.842	75.69
Grain yield	SH.N0	4MG-EEA	0.220	95.22
	SH.N50	3MG-CEA	0.910	43.57
	SH.N100	4MG-EEA	2.190	83.09
	WH.N50	4MG-EEA	1.262	79.42
	WH.N100	4MG-EEA	0.987	86.56
	All env.	4MG-EEA	0.048	96.18
Grain protein content	SH.N0	3MG-AI	0.068	38.61
	SH.N50	4MG-EEA	0.149	84.92
	SH.N100	3MG-AI	0.256	49.22
	WH.N50	2MG-ER	0.168	25.68
	WH.N100	2MG-AE	0.197	25.73
	All env.	3MG-AI	0.022	35.75

## Data Availability

The data presented in this study are available on request from the corresponding author. The data are not publicly available due to privacy.

## Supplementary Materials

The following supporting information can be downloaded at https://www.mdpi.com/article/10.3390/plants13010017/s1. Table S1: Meteorological data of the field trials; Table S2: Finlay-Wilkinson regression coefficient for all traits. File S1: Segregation analysis of grain yield and yield-related traits.
