# Supplementary material for: Phenotypic Plasticity of Yield and Yield-Related Traits Contributing to the Wheat Yield in a Doubled Haploid Population
Source: Plants (Basel). 2023 Dec 20;13(1):17. doi: 10.3390/plants13010017 (PMC10780773; doi:10.3390/plants13010017)
Supplement: Supplementary file 1 [file plants-13-00017-s001.zip › Supplementary table.pdf]

Table S1: Meteorological data of the field trials

A) Daily rainfall in Shenton Park in 2016 (Units = mm)

	**Jan**	**Feb**	**Mar**	**Apr**	**May**	**Jun**	**Jul**	**Aug**	**Sep**	**Oct**	**Nov**	**Dec**
**1st**	0	0	0	0	0	0	0	0.4	0.4	6.9	0	0
**2nd**	0	0	0	0	0	0	0	0	0.2	5.2	0	0
**3rd**	0	0	0	0	0.4	0	0	0	0	7.6	0	0
**4th**	0	0	0	0	0	6.0	0.4		0	0	0	0
**5th**	0	0	0	0	0	0	0	8.02	0	0	0	0
**6th**	0	0	0	0		0	0	0	0	0	0	0
**7th**	0	0	0	0	19.42	23.0	0	4.0	0	7.0	0	0
**8th**	0	0	0	0	1.4	0	11.2		8.8	10.0	0	0
**9th**	0	0	0	0	0	0	32.2	11.82	0	5.0	0	0
**10th**	0	0	0	11.8	0		30.0	4.2	6.0	0	0	0
**11th**	0	0	0	0	0	15.02	3.0		0	0	1.2	0
**12th**	0	0	0	3.4	0	0	0	11.22	0	0	0	4.8
**13th**	0	0	0	5.2	0	28.4	0	0	0	0	0	0
**14th**	0	0	0	0	3.4	0.2	0	0	0	1.2	0	0
**15th**	0	0	0	0	0	0	0	0	0	4.2	0	0
**16th**	0	0	0	0	0.4	0	0.6	5.6	9.8	1.8	0	0
**17th**	0	0	0	0	0	0		1.4	0	0	7.2	0
**18th**	1.4	0	0	1.4	4.8	0	32.42	18.0	0	0	0	0
**19th**	0	0	0	27.2	0.2	0	0	1.0	16.6	0	0	0
**20th**	0.4	0	0.4	0	0.2	7.8	0	3.6	0	0	0	0
**21st**	0	0	0	0		3.0	22.0	2.2	0	0	0	0
**22nd**	0	0.4	0	0		2.2		8.4	0	0	0	0
**23rd**	0	0	0	0	10.4	3.0	1.62	0	9.6	0	0	0
**24th**	0	0	0	0	29.2	0	1.8	0	0	0	0	0
**25th**	0	0	0	1.8	0.8		0	0	19.0	0	0	0
**26th**	0	0	11.0	28.0	1.4	10.02	0	0	0	0	0	0
**27th**	0	0	18.0	2.0	0	0	0	38.0	2.2	0	0	0
**28th**	0	0	0	1.3	4.4	0.8	0	7.0	7.4	0	0	0
**29th**	0	0	0	1.2	0	17.6	0	0.4	0	0	0	0
**30th**	0		0	5.8	0	0.8	0.5	0	1.2	0	0	0
**31st**	0		0		0.2		3.4	7.8		0		0

B) Daily temperature in Shenton Park in 2016 (Units = °C)

	Jan	Feb	Mar	Apr	May	Jun	Jul	Aug	Sep	Oct	Nov	Dec
**1st**	34.9	21.8	23.7	23.8	19.1	21.0	18.8	16.2	15.8	16.3	22.0	23.5
**2nd**	34.5	23.9	25.6	26.6	20.5	21.4	22.0	17.9	17.5	17.4	26.1	23.6
**3rd**	33.0	29.6	24.7	28.6	21.9	23.1	16.5	18.3	18.0	19.2	20.3	22.9
**4th**	29.5	33.9	24.5	25.2	22.3	17.7	14.4	15.6	20.3	20.4	26.2	21.8
**5th**	25.8	34.1	27.0	22.0	21.4	18.2	17.2	16.5	17.7	21.3	31.9	22.2
**6th**	33.9	27.8	29.8	23.9	20.4	17.8	17.9	18.0	19.1	20.3	23.1	23.5
**7th**	39.8	37.6	33.2	24.8	17.9	13.8	17.6	17.9	17.5	22.2	19.8	23.1
**8th**	39.1	42.8	32.9	28.1	16.6	15.2	17.6	14.9	17.2	18.6	21.1	28.4
**9th**	31.4	38.3	33.7	23.0	20.0	16.7	14.3	17.4	18.7	17.8	20.9	33.8
**10th**	25.9	33.6	26.2	27.1	22.0	18.7	16.3	17.8	18.8	18.1	21.2	36.2
**11th**	27.1	28.3	25.8	23.9	23.3	19.8	15.9	19.9	16.6	24.8	20.6	25.1
**12th**	24.8	26.2	29.8	25.1	23.4	21.5	14.7	17.2	17.6	33.4	21.7	23.7
**13th**	30.8	28.3	39.6	23.2	22.1	18.4	16.3	18.4	17.3	22.7	28.6	22.6
**14th**	34.9	31.3	40.7	23.1	21.7	18.8	18.6	19.6	18.8	21.1	37.5	27.8
**15th**	35.8	34.1	27.2	26.6	22.4	17.4	20.6	19.5	18.7	17.2	34.8	31.8
**16th**	36.6	39.1	24.2	32.3	21.0	18.2	19.9	19.4	16.1	18.6	20.3	27.5
**17th**	26.9	30.6	28.2	22.5	21.3	17.1	19.0	17.1	17.9	19.3	22.1	28.6
**18th**	23.4	30.9	29.9	22.8	21.5	20.0	16.0	14.8	17.5	19.9	27.0	23.1
**19th**	24.2	31.4	26.1	22.3	19.6	19.4	18.4	16.0	15.0	19.9	22.9	24.0
**20th**	25.3	31.5	28.3	22.3	21.5	17.6	17.6	17.6	19.1	18.0	21.9	33.3
**21st**	26.4	26.2	29.4	25.2	18.4	16.3	15.1	18.9	19.1	21.4	22.0	42.0
**22nd**	28.8	24.6	27.0	26.7	20.1	18.1	15.3	15.4	18.1	28.1	26.2	26.5
**23rd**	33.8	23.8	23.6	30.4	20.6	16.6	17.3	16.9	15.6	25.8	30.4	29.8
**24th**	32.2	22.6	27.1	24.8	19.4	17.7	14.5	16.8	17.2	20.4	32.0	25.5
**25th**	32.2	26.9	33.6	20.6	19.9	16.3	16.5	18.9	16.7	19.3	34.5	28.8
**26th**	31.0	34.8	22.9	21.5	19.7	17.8	18.0	21.2	18.0	20.6	36.8	27.1
**27th**	32.5	32.6	23.9	19.2	21.2	17.9	19.5	17.0	19.0	21.1	24.7	22.4
**28th**	34.7	25.8	25.5	21.3	19.8	17.6	20.3	15.7	15.0	21.1	25.7	21.9
**29th**	37.8	25.9	27.2	21.7	18.9	18.7	20.9	16.7	17.4	20.3	23.7	21.8
**30th**	27.0		27.3	19.1	19.6	17.5	19.4	17.4	19.1	19.4	23.2	22.5
**31st**	22.9		24.0		19.4		15.4	16.0		19.4		24.1

C) Daily rainfall in Wongon Hill in 2016 (Units = mm)

	**Jan**	**Feb**	**Mar**	**Apr**	**May**	**Jun**	**Jul**	**Aug**	**Sep**	**Oct**	**Nov**	**Dec**
**1st**	35.5	24.5	31.2	27.8	18.0	20.5	17.7	13.8	16.02		28.5	28.8
**2nd**	39.5	26.8	36.0	29.8	21.8	20.8	19.2	16.5	16.5	19.83	33.5	33.5
**3rd**	39.0	30.0	34.2	34.0	20.5	23.5	16.5	17.2	18.2	20.5	32.0	27.5
**4th**	35.5	33.5	29.6		22.0	16.0	12.8	14.5	23.8	23.5		27.5
**5th**	35.5	36.2	29.4	29.52	22.5	17.5	16.0	13.5	22.5	27.5	40.02	29.5
**6th**	38.5	37.9	31.0	22.5	21.8	16.5	16.5	19.0	21.8	25.5	32.0	31.5
**7th**	41.8	39.2	32.0	24.5	18.0	12.5	16.0	15.5	16.5	28.2	27.0	29.5
**8th**	36.2	40.5	34.0	22.8	14.8	12.7	16.5	15.0	17.5	18.5	30.0	30.5
**9th**	32.8	42.8	37.9	22.5	17.5	15.5	12.5	15.3	18.5	17.5		38.0
**10th**	32.0	44.2	38.5	29.0	22.0	17.9	15.5	16.8	19.5	20.5	24.52	41.5
**11th**	31.5	44.7		20.0	25.8	18.1	15.5	18.2	15.5	27.0	23.0	39.0
**12th**	31.9	39.0	37.72	24.0	26.5	19.8	11.5	15.2	10.0	33.0	26.4	25.0
**13th**	30.2	31.8	38.5	27.0	22.7	17.5	14.0	17.5	15.5	33.2	31.0	25.0
**14th**	33.0	31.5		24.5	20.8	15.5	16.0	25.0	18.1	23.5	36.0	30.0
**15th**	36.8	34.0	38.12	25.2	22.0	15.0	20.3	19.0	19.5	17.5	39.0	35.0
**16th**	38.0	38.8	28.0	32.3	20.0	16.8	19.8	18.8		21.5	24.0	
**17th**	38.0	39.5	25.8	27.5	21.0	16.5	17.5	19.0	17.02	23.5	25.4	33.52
**18th**	25.8	31.8	24.5	22.0	22.5		14.0	14.8	19.0	25.0		32.8
**19th**	21.5	31.7	29.0	22.8	17.0	19.52	14.8	14.3	15.5	22.5		31.5
**20th**	25.5	33.9	32.0	22.5	21.0	16.2	16.0	17.5	19.5	19.5	36.03	35.4
**21st**	28.2		33.0	23.8	16.0	14.5	13.8	17.5	24.5		26.2	41.5
**22nd**	32.5	31.52	23.4	26.3	17.0	15.8	13.1	14.8	19.5		30.5	34.8
**23rd**	37.5	27.0	26.0	28.5	18.5	14.5	15.5	13.5		35.23	33.0	37.8
**24th**	38.2	25.8	29.0	31.0	18.5	15.3	12.8	15.5		27.0	34.0	
**25th**	34.5	31.0	34.0	23.5	17.5	15.5	10.5	19.0	16.53	22.5	35.5	
**26th**	31.3	38.0	21.3	21.0	18.2	15.8	16.0	20.5	19.0	26.5	38.8	
**27th**	31.9	40.3	18.2	18.2	19.4	16.5	17.2	16.1	18.0	27.8	38.2	24.0
**28th**	33.5		21.0	20.0	19.8	20.0	22.0	15.5	15.2	27.0	36.0	26.8
**29th**	37.0	35.02	25.5	20.9	18.0	18.5	22.1	15.0	20.8	22.5	32.0	30.0
**30th**	33.0		28.5	17.5	17.0	15.5	18.7	17.0		23.2	32.5	29.5
**31st**	27.0		31.5		18.8		15.0			26.5		29.1

D) Daily temperature in Shenton Park in 2016 (Units = °C)

	Jan	Feb	Mar	Apr	May	Jun	Jul	Aug	Sep	Oct	Nov	Dec
**1st**	35.5	24.5	31.2	27.8	18.0	20.5	17.7	13.8	16.02		28.5	28.8
**2nd**	39.5	26.8	36.0	29.8	21.8	20.8	19.2	16.5	16.5	19.83	33.5	33.5
**3rd**	39.0	30.0	34.2	34.0	20.5	23.5	16.5	17.2	18.2	20.5	32.0	27.5
**4th**	35.5	33.5	29.6		22.0	16.0	12.8	14.5	23.8	23.5		27.5
**5th**	35.5	36.2	29.4	29.52	22.5	17.5	16.0	13.5	22.5	27.5	40.02	29.5
**6th**	38.5	37.9	31.0	22.5	21.8	16.5	16.5	19.0	21.8	25.5	32.0	31.5
**7th**	41.8	39.2	32.0	24.5	18.0	12.5	16.0	15.5	16.5	28.2	27.0	29.5
**8th**	36.2	40.5	34.0	22.8	14.8	12.7	16.5	15.0	17.5	18.5	30.0	30.5
**9th**	32.8	42.8	37.9	22.5	17.5	15.5	12.5	15.3	18.5	17.5		38.0
**10th**	32.0	44.2	38.5	29.0	22.0	17.9	15.5	16.8	19.5	20.5	24.52	41.5
**11th**	31.5	44.7		20.0	25.8	18.1	15.5	18.2	15.5	27.0	23.0	39.0
**12th**	31.9	39.0	37.72	24.0	26.5	19.8	11.5	15.2	10.0	33.0	26.4	25.0
**13th**	30.2	31.8	38.5	27.0	22.7	17.5	14.0	17.5	15.5	33.2	31.0	25.0
**14th**	33.0	31.5		24.5	20.8	15.5	16.0	25.0	18.1	23.5	36.0	30.0
**15th**	36.8	34.0	38.12	25.2	22.0	15.0	20.3	19.0	19.5	17.5	39.0	35.0
**16th**	38.0	38.8	28.0	32.3	20.0	16.8	19.8	18.8		21.5	24.0	
**17th**	38.0	39.5	25.8	27.5	21.0	16.5	17.5	19.0	17.02	23.5	25.4	33.52
**18th**	25.8	31.8	24.5	22.0	22.5		14.0	14.8	19.0	25.0		32.8
**19th**	21.5	31.7	29.0	22.8	17.0	19.52	14.8	14.3	15.5	22.5		31.5
**20th**	25.5	33.9	32.0	22.5	21.0	16.2	16.0	17.5	19.5	19.5	36.03	35.4
**21st**	28.2		33.0	23.8	16.0	14.5	13.8	17.5	24.5		26.2	41.5
**22nd**	32.5	31.52	23.4	26.3	17.0	15.8	13.1	14.8	19.5		30.5	34.8
**23rd**	37.5	27.0	26.0	28.5	18.5	14.5	15.5	13.5		35.23	33.0	37.8
**24th**	38.2	25.8	29.0	31.0	18.5	15.3	12.8	15.5		27.0	34.0	
**25th**	34.5	31.0	34.0	23.5	17.5	15.5	10.5	19.0	16.53	22.5	35.5	
**26th**	31.3	38.0	21.3	21.0	18.2	15.8	16.0	20.5	19.0	26.5	38.8	
**27th**	31.9	40.3	18.2	18.2	19.4	16.5	17.2	16.1	18.0	27.8	38.2	24.0
**28th**	33.5		21.0	20.0	19.8	20.0	22.0	15.5	15.2	27.0	36.0	26.8
**29th**	37.0	35.02	25.5	20.9	18.0	18.5	22.1	15.0	20.8	22.5	32.0	30.0
**30th**	33.0		28.5	17.5	17.0	15.5	18.7	17.0		23.2	32.5	29.5
**31st**	27.0		31.5		18.8		15.0			26.5		29.1

Table S2**:** Finlay-Wilkinson regression coefficient for all traits

Genotypes	Plasticity of					
	**Thousand kernel weight**	**Seed length**	**Spikelet number per spike**	**Seed number**	**Grain yield**	**Grain protein content**
DH001	1.82	0.83	1.40	0.89	0.61	1.29
DH002	1.35	0.97	0.74	1.00	1.10	1.00
DH003	0.44	1.35	1.60	1.18		
DH004		1.43	1.26	1.13		
DH005	1.89	0.98	1.97	1.04	1.20	0.92
DH006	1.37					
DH007				1.19	0.98	
DH008	1.75	1.34	2.94	1.11	0.89	0.92
DH009	1.51	0.69			0.99	
DH010	0.02	2.07	3.63	1.17	1.23	0.97
DH011	1.25	1.53	5.17	0.92	1.05	1.11
DH012	2.70	0.06	-1.24	1.01		
DH013	0.74	1.24	3.71	1.35	1.26	0.86
DH014	0.78	1.05	0.47	0.82		0.90
DH015			-1.39	1.11	1.12	
DH016	1.12	0.95	0.97	0.56	0.76	
DH017	2.43	0.27	1.82	0.85		0.85
DH018	0.49	1.08	1.05	1.03	0.99	
DH019	0.08	1.08	-1.20	0.76	1.05	

DH020		0.36	1.65	0.86		0.74
DH021	0.82	0.78	-2.05	0.59	0.96	1.10
DH022	0.28	0.93	3.06	0.89	0.69	1.02
DH023	1.94					
DH024			2.18	1.19	1.24	
DH025	0.46	0.98	2.28	1.35	0.92	
DH026	2.70	0.83	-0.35	1.24	1.22	0.76
DH027	-0.40	0.91	0.17	1.04		0.89
DH028	-0.97					
DH029			0.59	0.78	0.69	
DH030	2.25	0.53	0.59	0.65		
DH031		0.95	1.48	1.06	0.70	
DH032	0.76	1.09	3.34	1.19	1.07	1.13
DH033	2.10	0.07	-2.88	-0.17	0.82	0.81
DH034	0.44	0.98	3.13	1.04	0.83	
DH035		1.20	1.04	0.89		0.96
DH036	0.31				0.73	
DH037	2.78	1.30	0.52	1.39		0.96
DH038	0.89	0.80	2.05	0.60		0.93
DH039	0.60	1.05	-0.23	0.68	0.88	0.91
DH040		1.06	3.10	1.18	1.23	1.00
DH041	1.33	0.72	0.30	0.66	0.94	1.16
DH042	2.42	1.30	1.24	0.90	1.32	1.04
DH043	0.24	0.79	1.63	1.44	0.95	1.04
DH044	0.72	0.79	0.91	0.87	0.91	0.86

DH045	0.32					
DH046	2.72	1.15	-0.17	1.14	0.99	1.12
DH047	1.86	0.68	-0.54	0.78	0.63	1.00
DH048	0.30	0.76	1.91	1.44	1.08	0.74
DH049	1.60	1.52	1.02	1.12	1.18	1.07
DH050	0.72	0.64	-0.93	0.65	0.89	0.83
DH051	0.48	1.23	2.28	1.21	1.38	1.14
DH052	1.89	0.77	-0.63	1.05		0.94
DH053	0.10	1.35	0.99	0.65	1.25	1.05
DH054	0.59				0.62	
DH055	2.27	0.91	0.01	0.76	0.84	
DH056		1.20	2.01	1.10		0.97
DH057	0.72	0.57	-1.10	0.94	1.18	1.02
DH058	1.53	1.84	3.18	1.56	0.92	
DH059						0.97
DH060	1.22	0.91	0.42	0.97		0.90
DH061		1.40	0.81	0.97	0.90	0.71
DH062	-0.60	0.97	-0.98	1.37	1.14	0.84
DH063	0.76	1.53	-1.29	0.72		0.97
DH064	1.63	1.49	-0.46	0.59	0.69	1.66
DH065	-1.19	0.68	2.39	1.11	0.92	
DH066	1.89					
DH067						0.94
DH068	0.31	0.26	-1.47	0.86	1.16	0.92
DH069	1.13					

DH071			-0.04	0.60	1.01	1.12
DH072	0.87		-0.86	0.66	0.93	1.02
DH073	-0.73	0.57	2.22	0.89	0.70	1.13
DH074		1.23	-0.96	0.60		1.00
DH075	0.96	0.81	0.69	0.98	0.86	1.00
DH076	2.12	0.98	-0.70	1.04	0.59	
DH077	-0.03				1.12	1.13
DH078	0.96					0.73
DH079	1.79	1.45	4.25	1.20	0.89	
DH080	0.81	0.19	-2.03	0.33		1.10
DH081	0.92					
DH082			-0.01	1.01		1.24
DH083	0.55	0.93	-0.17	1.09	1.05	
DH084	0.74	1.14	2.81	0.99	0.87	0.95
DH085	-0.65	1.47	2.22	1.55	1.11	
DH086	-0.46	1.20	1.55	1.02	0.72	
DH087	0.78	1.09	1.40	0.93	1.24	0.85
DH088	-0.33	1.31	0.23	1.07	1.28	0.94
DH089	0.52	0.92	1.84	0.90	0.72	
DH090	0.45	1.03	-0.01	0.95	0.90	
DH091		2.16	1.78	1.59		
DH092	2.36					
DH093	1.94	0.84	0.73	0.99		0.79
DH094	1.22	0.98	0.55	0.95	0.90	0.87
DH095	1.28	1.04				

DH096	-0.19		3.29	1.44	0.87	1.30
DH097	2.91	1.44	0.98	1.11		1.25
DH098	3.02	0.76	3.60	1.04	0.95	1.02
DH099	2.16	1.01	0.81	0.87	0.81	
DH100	0.40	1.21	1.00	0.96		1.26
DH101	1.57	0.23	0.63	1.00	0.85	
DH102	2.49	0.53	1.15	1.11	0.99	
DH103	0.79					
DH104			0.25	1.10	1.28	
DH105	-0.18	0.85				0.89
DH106	1.43					
DH107					1.42	
DH108	2.38				1.23	
DH109		1.49	2.51	1.29		
DH110	0.01	1.08	-0.96	1.28	0.97	
DH111		1.15	1.97	1.26	1.22	1.08
DH112	2.17	0.94	2.53	1.03	1.06	1.20
DH113	1.07	1.36	2.11	1.56	1.14	0.79
DH114	-0.15	1.09	1.39	1.20	1.22	0.96
DH115	-0.92	1.04	-0.80	0.85	0.70	
DH117	0.62	1.21	1.00	1.44		0.86
DH118	0.55	0.85	0.24	0.79	0.72	1.11
DH119	-0.24	0.99				0.85
DH120	1.69		0.44	1.17	1.22	
DH121	1.78	0.55	0.18	0.87	1.08	1.15

DH122	-0.49					
DH123			0.85	1.50	1.52	0.93
DH124	1.19	0.72	-0.50	0.97	1.04	1.34
DH125	2.19	1.82	1.73	1.25	1.33	1.12
DH126	1.46	0.49	-0.30	0.43		0.99
DH127	-0.45					
DH128			0.52	1.36	1.01	0.98
DH129	1.51	0.94	3.26	0.67	0.69	1.01
DH130	1.39	1.58	0.17	1.45	1.62	0.89
DH131	1.79	0.89				
DH132	-0.39					
DH133			3.30	1.06		1.28
DH134	3.11	0.91	1.81	0.75	0.93	0.90
DH135		0.86	-1.70	0.88		
DH136	1.31	0.58	1.30	1.29	1.31	0.96
DH137	-0.15	1.19	0.74	0.79	0.91	
DH138						1.08
DH139	0.20	0.85	1.57	0.88		0.93
DH140	1.17	1.08	-0.37	1.11	0.87	1.22
DH141	2.59	0.69	0.12	0.61	1.23	0.77
DH142	0.40	1.21	3.62	1.19	1.15	0.98
DH143	1.59	1.16	0.44	1.26	1.03	0.94
DH144	-0.28	0.86	-0.15	0.46	0.63	1.06
DH145		0.48	0.69	0.47		1.04
DH146	1.05	1.07	2.83	1.10		0.97

DH147	-1.07				1.04	0.94
DH148	-1.05	1.14	3.09	1.02		0.96
DH149	1.09	0.73	-0.10	1.16	0.98	0.91
DH150		1.46	4.17	1.20		1.29
DH151	3.25					
DH152	1.46					
